# Extracellular vesicle-derived miR-760 as a novel promising candidate biomarker differentiating stable RRMS from SPMS

**DOI:** 10.1038/s41598-026-35189-y

**Published:** 2026-01-14

**Authors:** Karina Wasilewska, Angela Dziedzic, Shamundeeswari Anandan, Elżbieta Miller, Łukasz Łaczmański, Radosław Zajdel, Sylwia Michlewska, Dorota Kujawa, Marta Gancarek, Justyna Raczkowska, Lidia Włodarczyk, Patrycja Nowak, Joanna Saluk

**Affiliations:** 1 Faculty of Biology and Environmental Protection, Department of General Biochemistry, University of Lodz, Pomorska 141/143, Lodz, 90-236 Poland; 2 Department of Clinical Medicine, University of Bergen, 5021 Bergen, Norway; 3 Neuro-SysMed, Department of Neurology, Haukeland University Hospital, 5021 Bergen, Norway; 4 Department of Neurological Rehabilitation, Medical University of Lodz, Milionowa 14, Lodz, 93-113 Poland; 5 Ludwik Hirszfeld Institute of Immunology and Experimental Therapy, Laboratory of Genomics and Bioinformatics, Polish Academy of Sciences, Weigla 12, Wroclaw, 53-114 Poland; 6 Department of AI in HealthCare Monitoring, Medical University of Lodz, Lodz, 90-645 Poland; 7 Faculty of Biology and Environmental Protection, Laboratory of Microscopic Imaging and Specialized Biological Techniques, University of Lodz, Banacha 12/16, Lodz, 90-237 Poland

**Keywords:** Multiple sclerosis, Extracellular vesicles, MiRNA, MiR-760, Biomarkers, Neuroinflammation, Neurodegeneration, Biomarkers, Diseases, Molecular biology, Neurology, Neuroscience

## Abstract

Multiple sclerosis (MS) is a chronic autoimmune disease of the central nervous system with heterogenous clinical course, lacking non-invasive biomarkers for phenotype differentiation. This study aimed to explore circulating extracellular vesicle (EV)-derived microRNA (miRNA) signatures and related molecular profiles capable of distinguishing stable relapsing-remitting MS (RRMS) from secondary progressive MS (SPMS). Plasma samples were collected from stable RRMS (*n* = 30), SPMS (*n* = 30), and healthy controls (HC) (*n* = 30), followed by total EVs isolation and characterization using transmission electron microscopy, dynamic light scattering, and flow cytometry. RNA was extracted from EVs, and miRNA profiles were analyzed via RNA sequencing and RT-qPCR. Cytokines and neuronal/astroglial injury biomarkers were quantified using the BioPlex system and ELISA. Functional enrichment and network analyses of miRNA targets were performed, alongside logistic regression modeling to explore potential distinguishing features. Four EV-derived miRNAs (miR-760, miR-98-5p, miR-301a-3p, miR-223-3p) showed significant differences (*p* < 0.05) between stable RRMS and SPMS. An integrative model combining miRNAs with fibroblast growth factor (FGF) basic protein enabled accurate phenotypic differentiation (AUC = 0.942). miR-760 showed the strongest distinctive capacity for stable RRMS. Additionally, miR-98-5p was markedly up-regulated in both stable RRMS and SPMS compared to HC. Network analysis of miRNA targets suggested distinct immunoregulatory patterns across MS phenotypes. Plasma EV-derived miRNAs—particularly miR-760, and miR-98-5p—showed potential as molecular indicators associated with disease phenotype in MS. Integrating EV-miRNA profiling with protein markers support efforts toward more precise stratification of MS patients. Further studies in independent cohorts and functional validation are warranted before clinical translation.

**Supplementary Information:**

The online version contains supplementary material available at 10.1038/s41598-026-35189-y.

## Introduction

Multiple sclerosis (MS) is a chronic immune-mediated disorder affecting approximately 2.8 million people worldwide as of 2020^1^. It primarily targets the central nervous system (CNS), leading to focal and diffuse neuroinflammatory damage in the brain and spinal cord. The disease is driven by a sustained inflammatory response involving CD4^+^ and CD8^+^ T cells, B cells, and other immune mediators reactive against myelin antigens^2^.

Clinically, MS manifests in heterogeneous phenotypes. Approximately 85% of patients initially present with a clinically isolated syndrome (CIS) that progresses to a relapsing-remitting course (RRMS), while a subset eventually transitions to secondary progressive MS (SPMS). A smaller group exhibits primary progressive MS (PPMS), characterized by insidious neurological decline from onset^3^. The variability in radiological, histopathological, and clinical presentation, along with differential drug responsiveness, complicates both diagnosis and disease monitoring^4^.

Despite advancements in MS diagnostics over the past decade, it still relies heavily on clinical assessment, necessitating careful differentiation from alternative conditions. The introduction of AQP4-IgG and MOG-IgG assays has improved the distinction between neuromyelitis optica spectrum disorder (NMOSD) and myelin oligodendrocyte glycoprotein antibody-associated disease (MOGAD) from MS. However, the absence of MS-specific biomarkers remains a major clinical challenge^5^. Routine blood tests, such as neurofilament light chain (NfL), which is not entirely disease-specific, have limited diagnostic value, increasing the risk of misdiagnosis^5,6^.

Emerging evidence suggests that extracellular vesicles (EVs) play a potential role in the clinical medicine, as reservoirs of biomarkers that reflect the pathological state in immune and neurodegenerative disease^7,8^. EVs, as defined by the International Society of Extracellular Vesicles (ISEV), encompass two major types of vesicles – exosomes and ectosomes (microvesicles)^9^. Released by parental cells, EVs mediate intercellular communication by transferring bioactive molecules, including nucleic acids, lipids, and proteins. In MS, EVs are implicated in antigen presentation, blood-brain barrier (BBB) disruption, lymphocyte activation, and CNS infiltration, reflecting disease pathology^10^. Among EV-associated candidates for biomarkers, microRNAs (miRNAs) are of particular interest due to their stability in body fluids and their regulatory role in post-transcriptional gene expression^11^. Several miRNAs, including miR-155, miR-146a, and miR-181c, have been identified as potential MS biomarkers, correlating with disease activity, relapse risk, and expanded disability status scale (EDSS) scores^12–15^. However, the clinical applicability of EV-miRNA signatures in MS remains uncertain due to methodological heterogeneity, emphasizing the need for further validation, which may ultimately lead to the development of standardized analytical approaches^16^. Further robust and validated data are essential before implementation of standarized biomarkers to complement clinical approaches and support accurate MS diagnosis or phenotype stratification.

Therefore, based on previously published data and our novel RNA-seq findings, we aim to refine the candidate pool of EV-associated miRNAs potentially relevant to MS pathophysiology in stable RRMS and SPMS. Using a multi-omic approach combining miRNA expression profiling, protein marker quantification, and integrative bioinformatics, we aimed to identify molecular signatures associated with disease activity and explore their functional relevance in the context of MS progression.

## Materials and methods

### Sample collection and preparation

Peripheral blood samples were collected via venipuncture between 8:00 and 9:00 a.m. in Sarstedt tubes (Nümbrecht, Germany) containing citrate phosphate dextrose adenine (CPDA)-1 as an anticoagulant. Plasma was separated by centrifugation at 235 × *g* for 12 min. at 25 °C, aliquoted, and stored at -80 °C until further analysis.

A total of 60 patients with MS were recruited from the Department of Rehabilitation, Neurological Rehabilitation Division, III General Hospital in Lodz, Poland. MS diagnosis was confirmed based on the 2017 McDonald criteria^17^, and disease phenotype classification followed Lublin et al.^18^. Additional inclusion criteria required absence of current or recent (< 6 months) disease-modifying therapies (DMTs) or other immunomodulatory treatment. RRMS patients were assessed within 3 weeks after completing a standard 5-day course of intravenous methylprednisolone (1000 mg/day). SPMS patients were not receiving corticosteroids or DMTs at the time of sampling. All participants provided written informed consent (Research Bioethics committee with resolution No. 3/KBBN- UŁ/IV/2018) and completed a detailed medical questionnaire. Clinical assessments included neurological examinations, magnetic resonance imaging (MRI) to evaluate grey matter pathology and white matter lesion volume, and disability assessment using the EDSS.

Exclusion criteria for MS patients included the use of medications affecting platelet biology, disease-modifying therapies (e.g., interferon-β, glatiramer acetate, natalizumab), hormones, corticosteroids, or immunomodulators; recent infections (≤ 4 weeks); comorbid neurological or psychiatric disorders; diabetes mellitus; myocardial infarction; pregnancy; or breastfeeding.

A healthy control group (HC, *n* = 30) was recruited from the Laboratory Diagnostics Center in Lodz, Poland. HC participants were age- and sex-matched to the MS cohort and confirmed to be free of MS, autoimmune diseases, neurodegenerative disorders, and acute or chronic inflammatory conditions. Additional exclusion criteria included pregnancy, breastfeeding, and medication use. Health status was verified through comprehensive medical evaluation, including routine hematological and inflammatory marker assessments.

### Bio-Plex multiplex immunoassay

Cytokine profiling was performed using the Bio-Plex Pro multiplex assay kit (cat. M500KCAF0Y; Bio-Rad Laboratories, Inc., USA) following the manufacturer’s instructions. This assay enabled the simultaneous quantification of the following 27 cytokines in a single sample: fibroblast growth factor (FGF) basic, eotaxin, granulocyte colony-stimulating factor (G-CSF), granulocyte-macrophage colony-stimulating factor (GM-CSF), interferon (IFN)-γ, interleukin (IL)-1β, IL-1ra, IL-2, IL-4, IL-5, IL-6, IL-7, IL-8, IL-9, IL-10, IL-12, IL-13, IL-15, IL-17 A, interferon gamma-induced protein (IP)-10, monocyte chemoattractant protein (MCP)-1, macrophage inflammatory protein (MIP)-1α, MIP-1β, platelet-derived growth factor (PDGF)-BB, regulated on activation, normal T expressed and secreted (RANTES), tumor necrosis factor (TNF)-α, and vascular endothelial growth factor (VEGF). Prior the analysis, the instrument was calibrated and validated to ensure optimal performance of fluidics and optics systems.

Plasma samples were thawed at 4 °C and kept on ice until use. Reagents were equilibrated to room temperature (RT) before the assay. To remove precipitates, samples were centrifuged at 10,000×*g* for 10 min at 4 °C and diluted 1:4 with the provided sample diluent buffer. Antibody-coupled beads were prepared, added to a 96-well microplate, and incubated with diluted plasma samples, standards, or quality controls for 1 h at RT on a shaker. After washing, biotin-labeled detection antibodies were added and incubated for 30 min at RT, followed by streptavidin-phycoerythrin conjugates for 10 min at RT, with washing steps between incubations. Finally, assay buffer was added, the plate was shaken to resuspend the beads, and fluorescence was measured using the Bio-Plex 200 System analyzer (Bio-Rad Laboratories, Inc., USA). Cytokine concentrations were determined using Bio-Plex Manager software (Bio-Rad Laboratories, Inc., USA) with standard curve interpolation. Additional data, including minimal expected concentration and CV% for each biomarker, have been provided in Supplementary Materials (Table S1).

### Measurement of neuronal/astroglial injury biomarkers concentration

Human enzyme-linked immunosorbent assay (ELISA) kits were applied to measure the plasma concentrations of NfL (cat. HEE038Hu; Cloud-Clone Corp., USA) and glial fibrillary acidic protein (GFAP) (cat. E-EL-H6093; Elabscience, USA). All measurements were performed in duplicate using the UV-Vis microplate reader SPECTROstar Nano system (BMG Labtech GmbH, Germany). Protein concentrations were calculated by comparing the optical density (OD) values of the samples to standard curves. Additional data, including lower limit of detection and CV%, have been provided in Supplementary Materials (Table S2).

### Extracellular vesicles isolation and characterization

Prior to EVs isolation, plasma samples (1 ml) were treated with RNase A (100 ng/ml) for 10 min. at 37 °C to remove unprotected circulating RNA^19^. EVs were then isolated using the Total Exosome Isolation Kit (plasma) (cat. 4484450; Invitrogen, USA) following the manufacturer’s protocol. The resulting pellet was resuspended in 200 µl of 1× phosphate-buffered saline (PBS) and stored at -20 °C for short-term preservation.

To assess the purity and morphology of the isolated EVs, nine randomly selected samples (three from each MS group and the HC group) were analyzed using transmission electron microscopy (TEM). EV samples were fixed with 2.5% glutaraldehyde and placed on 200-mesh carbon-coated copper grids (Polysciences, USA). The samples were then stained with 2% uranyl acetate (ACS Reagent, USA) and examined using a JEM-1010 transmission electron microscope (JEOL Ltd., Tokyo, Japan). Developed films were scanned using the Perfection V700 PHOTO scanner (Epson, Japan).

Dynamic light scattering (DLS) was used to assess the size distribution and homogeneity of extracellular vesicle population. The hydrodynamic diameter and polydispersity index (PDI) were measured using a Zetasizer Nano-ZS (Malvern Instruments Ltd., UK), 633 nm laser, 173° detection angle, in PBS (pH 7.4), 25 °C. EVs size was determined from three independent replicates, with the average calculated from five runs per sample.

To assess EV characteristics, 18 randomly selected samples (6 from each RRMS, SPMS, and HC group) were analyzed for EV surface markers using flow cytometry. EV-associated proteins CD63-FITC and CD81-Pacific Blue (Beckman Coulter, Brea, CA, USA) were detected by direct staining with fluorochrome-conjugated anti-human monoclonal antibodies. Samples were incubated at RT for 30 min in the dark before analysis on a BD FACSymphony A1 flow cytometer (Becton Dickinson, USA). Data were acquired and processed using BD FACSDiva software (v9.0.2).

### Total RNA isolation

Total RNA, including miRNA, was extracted from plasma-derived EVs using the Total Exosome RNA & Protein Isolation Kit (cat. 4478545; Invitrogen, USA) employing acid-phenol: chloroform for organic extraction according to the manufacturer’s protocol. Briefly, ethanol was added to the aqueous phase obtained from acid-phenol: chloroform extraction and passed through a glass-fiber filter cartridge, which immobilized the RNA. The filter was subsequently washed, and RNA, including the small RNA fraction, was eluted using a low ionic-strength buffer.

To monitor RNA recovery and reverse transcription efficiency, 10 pM of synthetic cel-miRNA-39 (5′-UCACCGGGUGUAAAUCAGCUUG-3′) was added to each sample before RNA isolation.

RNA concentration was assessed using the Agilent RNA 6000 Pico Kit and Agilent 2100 Bioanalyzer (Agilent Technologies, USA). Extracted RNA was stored at -80 °C until further processing for RNA sequencing.

### RNA sequencing

The screening RNA expression analysis was performed for 9 randomly selected samples (3 from each RRMS, SPMS, and HC group). RNA sequencing libraries were prepared using the QIAseq miRNA Library Kit (cat. 331502; Qiagen) following the manufacturer’s protocol. Libraries were sequenced on the Illumina NextSeq 500/550 platform using Mid Output kits (v2.5). Image processing, base calling, and demultiplexing were performed with NextSeq Control Software (Illumina, San Diego, CA, USA).

### Selected MiRNA expression analysis

Total RNA was reverse transcribed into complementary DNA (cDNA) using the TaqMan Advanced miRNA cDNA Synthesis Kit (cat. A28007; Applied Biosystems, USA) according to the manufacturer’s protocol. Undiluted cDNA was stored at -80 °C prior to quantitative real-time PCR (RT-qPCR) analysis.

TaqMan Advanced miRNA Assays (Applied Biosystems, USA) were applied to quantify each miRNA expression (Table 1). qPCR reactions were carried out using TaqMan Fast Advanced Master Mix (Applied Biosystems) following the manufacturer’s protocol. cDNA was diluted 1:10 in 0.1× TE buffer, and 5 µl of the diluted sample was used as the template in a total reaction volume of 20 µl.

Endogenous control genes for RT-qPCR were selected using the RefGenes^20^ tool within the Genevestigator^21^ database and validated experimentally to identify the most stably expressed reference gene under the study conditions. The stability of the selected reference gene (miR-451a) was estimated using the NormFinder algorithm^22^, by modeling within- and between-group variance components on RT-qPCR data expressed on a linear (RQ) scale. The overall stability value of 0.201 indicated high expression stability of miR-451a across the clinical groups. Endogenous control gene expression has been provided in Supplementary Materials (Fig. S1).

RT-qPCR was performed on the CFX96 Touch Real-Time PCR Detection System (Bio-Rad Laboratories, Inc., USA) under the following conditions: polymerase activation (20 s., 95 °C), denaturation (3 s., 95 °C), and extension (30 s., 60 °C) for 49 cycles. Fluorescence detection was recorded as cycle threshold (Ct) values (Supplementary Materials, Table S3).

miRNA expression levels were quantified using the comparative ∆∆Ct method. For each sample, the ∆Ct value was determined as the difference between the Ct value of the target miRNA and that of the endogenous reference gene (miR-451a). The mean ∆Ct of the HC group was used as the reference baseline. The ∆∆Ct value for each sample in the RRMS and SPMS groups was calculated as the difference between its ∆Ct and the mean ∆Ct of the HC group. Fold-change (FC) values were determined using the 2^−∆∆Ct^ and log_2_-transformed for better clarity of results presentation.

**Table 1:** MiRNA assays selected for RT-qPCR analysis.

Assay name	Mature miRNA Sequence	Assay ID
hsa-miR-451a	AAACCGUUACCAUUACUGAGUU	478107_mir
hsa-miR-223-3p	UGUCAGUUUGUCAAAUACCCCA	477983_mir
hsa-miR-98-5p	UGAGGUAGUAAGUUGUAUUGUU	478590_mir
hsa-miR-760 (corresponding to miR-760-3p)	CGGCUCUGGGUCUGUGGGGA	483112_mir
hsa-miR-16-5p	UAGCAGCACGUAAAUAUUGGCG	477860_mir
hsa-miR-23a-3p	AUCACAUUGCCAGGGAUUUCC	478532_mir
hsa-miR-146a-5p	UGAGAACUGAAUUCCAUGGGUU	478399_mir
hsa-miR-181c-5p	AACAUUCAACCUGUCGGUGAGU	477934_mir
hsa-miR-155-5p	UUAAUGCUAAUCGUGAUAGGGGUU	483064_mir
hsa-miR-326	CCUCUGGGCCCUUCCUCCAG	478027_mir
hsa-miR-301a-3p	CAGUGCAAUAGUAUUGUCAAAGC	477815_mir
hsa-miR-191-5p	CAACGGAAUCCCAAAAGCAGCUG	477952_mir

### MiRNA target genes and functional enrichment analysis

To identify functionally relevant target genes of the differentially expressed miRNAs (RRMS vs. SPMS), we filtered experimentally validated interactions from miRecords, miRTarBase, and TarBase databases using the multiMiR package and org.Hs.eg.db database in R (v4.4.2)^23^. miR-760 was selected for extended analyses based on its promising differential expression pattern (RRMS vs. SPMS). To obtain deepen results, we integrated experimentally validated and in silico predicted targets of miR-760, downloaded from miRWalk 3.0.

Gene ontology (GO) enrichment analysis was performed to identify associated biological processes (BP), cellular components (CC), and molecular functions (MF). Pathway enrichment analysis was conducted using Kyoto encyclopedia of genes and genomes (KEGG) pathway database^24,25^. For both overrepresentation analysis, the clusterProfiler package was used, applying Benjamini and Hochberg correction and a significance threshold of *p* < 0.05. Pathways were ranked based on GeneRatio (the proportion of input genes mapping to a given pathway) and adjusted p-values, and visualizations were generated using the enrichplot package^26^.

To investigate molecular differences between RRMS and SPMS, we integrated publicly available disease-associated genes with experimentally validated mRNA targets of miR-98-5p, miR-760, miR-301a-3p, and miR-223-3p. Disease-related genes were retrieved from the DisGeNET (v24.4) database^27,28^ by querying “secondary progressive multiple sclerosis” for SPMS and “multiple sclerosis relapse” and “multiple sclerosis exacerbation” for RRMS. The top 30 genes for each disease phenotype were selected from DisGeNET based on the highest disease-gene association (GDA) scores, which quantify the strength of the gene-disease relationship.

To identify group-specific regulatory interactions, we cross-referenced these disease-associated gene lists with our miRNA-target dataset. To enhance the specificity of target, genes common to both RRMS and SPMS were excluded, focusing on distinct molecular signatures differentiating the two phenotypes. The remaining miRNA-mRNA pairs were used to construct interaction networks for RRMS and SPMS in Cytoscape (v3.10.3).

For data visualizations ggplot2 package was applied^29^. Figures were edited using GIMP (v2.10.38).

### Statistical analysis

Statistical analyses were performed using STATISTICA Software (v13.3) (StatSoft; Tulsa, OK, USA). A p-value < 0.05 was considered statistically significant for all tests.

The Shapiro-Wilk test was employed to assess the normality of the distribution of the data. For non-normally distributed data, the U Mann-Whitney test was used for assessing the statistical significance of differences between two independent groups. The Kruskal-Wallis test was used while assessing the statistical significance of differences between three independent groups for non-normally distributed data. Following the Kruskal-Wallis test, false discovery rate (FDR) method of Benjamini and Hochberg was applied to perform multiple comparisons between the groups. Correlations were analyzed using Spearman’s rank test separately for RRMS and SPMS groups. To account for multiple testing, the Benjamini and Hochberg correction was applied.

To identify statistically significant variables, univariate statistical analysis was conducted using logistic regression. Variables identified as significant in the univariate analysis (*p* < 0.05) were included in a multivariate logistic regression model to assess the probability of specific clinical outcome (RRMS vs. SPMS). The model was developed using the stepwise forward method, excluding variables with high collinearity based on correlation analysis performed using Spearman’s rank correlation coefficient for non-normally distributed data. Sigma-restricted parameterization was applied in the analysis to stabilize coefficient estimates and ensure interpretable reference categories. The Hosmer-Lemeshow test was applied to assess the goodness-of-fit of the logistic regression model. A p-value > 0.05 in this test indicated adequate model fit, demonstrating consistency between predicted probabilities and observed outcomes.

Factor analysis was applied to identify clusters of interrelated variables. The principal component extraction method with Varimax rotation was used to reduce the number of variables to key factors, ensuring interpretability of the results.

Receiver operating characteristic (ROC) curve analysis was performed for selected variables to evaluate their ability to distinguish between clinical groups and confirmed via k-fold cross-validation. The area under the curve (AUC) was calculated as a measure of sensitivity and specificity, with 95% confidence intervals (CI).

## Results

### Study groups

A total of 60 MS patients (30 RRMS and 30 SPMS) and 30 HC were included in the study. Basic clinical characteristics, including age, gender, C-reactive protein (CRP) levels, erythrocyte sedimentation rate (ESR), disease duration, and EDSS scores, were collected. A summary of participant characteristics is presented in Table 2.

**Table 2:** Clinical features of study groups.

Clinical characteristics	HC (*n* = 30)	RRMS (*n* = 30)	SPMS (*n* = 30)
Age (years)	46.7 ± 11.88	43.9 ± 10.5	62.7 ± 7.7
Gender, female/male (%)	18/12 (60/40)	19/11 (63.3/36.7)	17/13 (56.7/43.3)
CRP ^a)^ (mg/L)	2.26 ± 2.46	10.21 ± 10.99	15.64 ± 28.89
ESR ^b)^ (mm/h)	9.0 ± 9.8	29.37 ± 15.11	33.3 ± 12.7
Disease duration (years)	N/A	10.9 ± 6.3	30.6 ± 8.7
EDSS ^c)^	N/A	5.0 ± 1.3	5.8 ± 0.5
Treatment status	N/A	≤ 3 weeks after 5-day IV methylprednisolone (1000 mg/day); no DMTs ^d)^	No DMTs; no corticosteroids

Parameters presented as mean ± SD. ^(a)^ CRP, C-reactive protein; ^(b)^ ESR, erythrocyte sedimentation rate; ^(c)^ EDSS, Expanded Disability Status Scale; ^(d)^ DMTs, disease-modifying therapies.

### Differentiated profile of inflammatory and neuronal/astroglial injury biomarkers in MS patients

Of the 27 cytokines measured using the multiplex immunoassay, eight (IL2, IL-5, IL-6, IL-7, IL-12, IL-15, PDGF-BB, VEGF) were excluded from further analysis due to concentrations falling below the detection limit. Additionally, two neuronal and astroglial injury biomarkers (NfL and GFAP, respectively) were analyzed.

Table 3 presents the median (± interquartile range, IQR) values for remaining 19 cytokines and two neuronal and astroglial injury biomarkers. The analysis revealed significant alterations in biomarkers concentration in both RRMS and SPMS patients compared to HC. The most pronounced increase was observed for GM-CSF, with a 5.92-fold elevation in RRMS and a 10.83-fold increase in SPMS. Among the most dysregulated cytokines, IL-1ra exhibited a 3.81-fold rise in RRMS and a 2.73-fold increase in SPMS. Similarly, MIP-1β (2.3-fold in RRMS, 2.65-fold in SPMS) and MIP-1α (2.54-fold in SPMS) showed marked elevation. Pro-inflammatory cytokines such as TNF-α (2.49-fold in RRMS, 2.5-fold in SPMS) and IL-8 (3.74-fold in RRMS) also demonstrated substantial up-regulation. Notably, the overall trend suggested a more pronounced increase in SPMS.

A total of 16 cytokines and neuronal injury biomarker (NfL) showed statistically significant differences across groups in the Kruskal-Wallis test and were further analyzed using the FDR method by Benjamini and Hochberg for RRMS and SPMS subgroups.

**Fig. 1:**
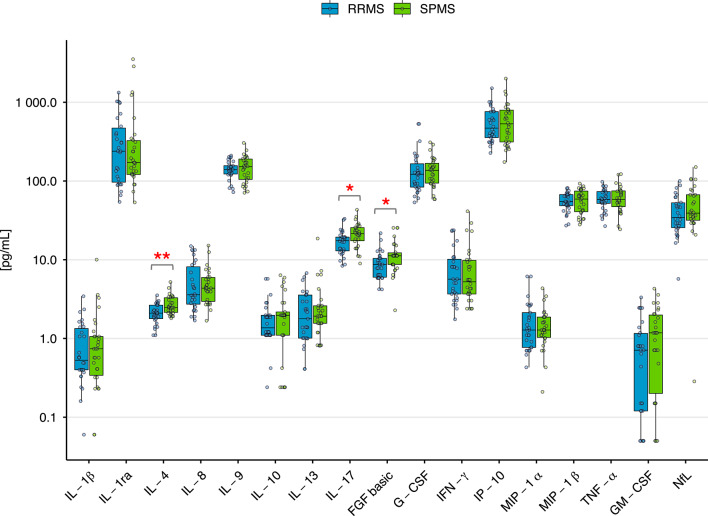
Differential concentration of selected plasma-derived cytokines between RRMS and SPMS patients. Plasma cytokine levels measured by Bio-Plex multiplex immunoassay and ELISA. Data are presented as median ± IQR. Statistical significance was determined using the Mann-Whitney U test followed by Benjamini-Hochberg FDR correction (*p < 0.05 and **p < 0.01).

Ultimately, three markers exhibited significant differences in concentration between the analyzed subgroups, with all showing increased mean concentrations in SPMS patients compared to RRMS (Fig. 1). IL-4, a cytokine regulating the immune response, promoting the differentiation of T helper (Th)2 cells was 1.27-fold elevated (*p* = 0.0044). IL-17, which contributes to inflammation by promoting the activation of Th17 cells, showed a 1.26-fold increase (*p* = 0.033). FGF basic, the growth factor involved in neuroprotection and myelination, exhibited a 1.33-fold increase (*p* = 0.01).

**Table 3:** Plasma cytokine profiles in RRMS and SPMS patients and HC.

Cytokine concentration	HC (*n* = 30) [median ± IQR]	RRMS (*n* = 30) [median ± IQR]	SPMS (*n* = 30) [median ± IQR]	*p*-value (Kruskal-Wallis)
IL-1b [pg/ml]	0.33 ± 0.35	0.57 ± 1.01	0.74 ± 1.27	0.0138
**IL-1ra [pg/ml]**	62.87 ± 19.96	**239.30 ± 324.7**	**171.6 ± 226.2**	< 0.0001
IL-4 [pg/ml]	1.33 ± 0.64	2.05 ± 0.91	2.54 ± 1.12	< 0.0001
**IL-8 [pg/ml]**	0.94 ± 0.8	**3.52 ± 4.66**	4.44 ± 3.98	< 0.0001
Il-9 [pg/ml]	62.97 ± 38.95	140.2 ± 38.7	151.8 ± 83.6	< 0.0001
IL-10 [pg/ml]	1.21 ± 0.79	1.38 ± 0.87	1.97 ± 2.4	0.0173
IL-13 [pg/ml]	1.2 ± 1.35	2.17 ± 2.56	2.01 ± 1.93	0.0458
IL-17 [pg/ml]	8.54 ± 3.39	17 ± 7.42	21.46 ± 8.73	< 0.0001
RANTES [pg/ml]	640.5 ± 399.2	1318 ± 1097.5	1285 ± 1229.5	< 0.0001
Eotaxin [pg/ml]	79.81 ± 54.62	73.81 ± 39.48	84.41 ± 41.06	> 0.05
FGF basic [pg/ml]	4.97 ± 4.42	7.81 ± 4.54	11.24 ± 4.4	< 0.0001
G-CSF [pg/ml]	52.14 ± 13.93	103.5 ± 55.35	139 ± 93.6	< 0.0001
IFN-g [pg/ml]	2.68 ± 1.23	5.67 ± 6.1	4.7 ± 6.78	< 0.0001
IP-10 [pg/ml]	322.1 ± 223.9	411.9 ± 444.8	563.8 ± 501.3	> 0.05
MCP-1 [pg/ml]	17.65 ± 8.69	16.65 ± 10.94	16.28 ± 11.15	> 0.05
MIP-1a [pg/ml]	0.52 ± 0.31	1.1 ± 1.16	1.32 ± 0.93	< 0.0001
**MIP-1b [pg/ml]**	22.26 ± 12.23	51.25 ± 22.27	**58.97 ± 31.99**	< 0.0001
TNF-a [pg/ml]	23.45 ± 24.53	58.43 ± 22.03	58.53 ± 29.78	< 0.0001
**GM-CSF [pg/ml]**	0.12 ± 0.43	**0.71 ± 1.11**	**1.18 ± 1.89**	0.0005
GFAP [pg/ml]	145.8 ± 70.9	170 ± 108.2	190.4 ± 114.1	> 0.05
NfL [pg/ml]	18.44 ± 17.6	34.38 ± 30.6	39.11 ± 38.2	< 0.0001

Bolded values are the top three with the highest fold change within each patient group compared to HC.

### Characterization of plasma-derived EVs

**Fig. 2:**
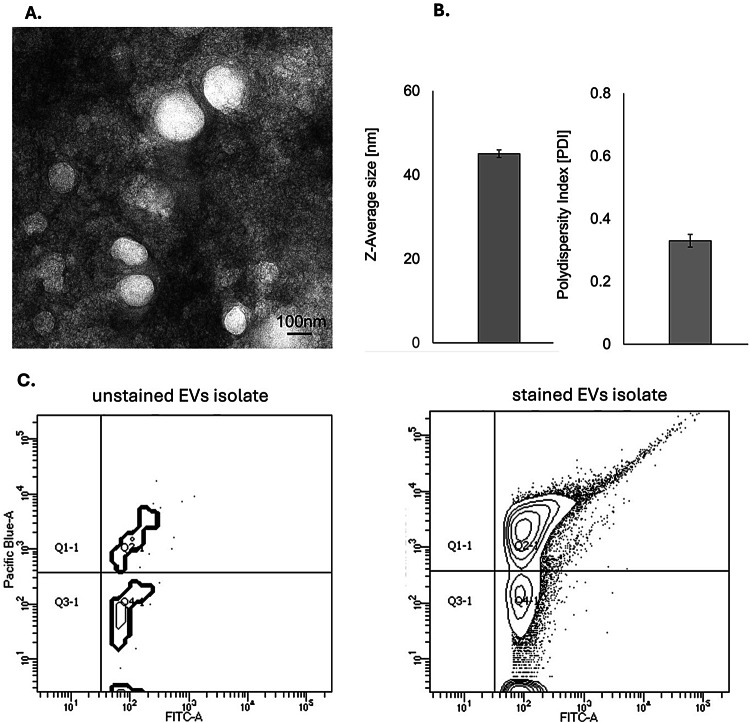
Characterization of plasma extracellular vesicles (EVs). (**A**) Representative transmission electron microscopy (TEM) image showing round, membrane-bound vesicles. Scale bar = 100 nm. (**B**) Size distribution and polydispersity index (PDI) of EVs measured by dynamic light scattering (DLS), presented as mean ± SEM. (**C**) Contour plot of EVs isolates stained with anti-CD63 FITC and anti-CD81 Pacific Blue and analyzed by flow cytometry. For the negative control unstained EVs isolates were used.

TEM images revealed a heterogeneous population of vesicles with predominantly spherical morphology and well-defined membrane boundaries (Fig. 2A). The DLS analysis further supported the structural integrity and purity of the sample, confirming a z-Average diameter of 45 nm and a PDI of 0.33, indicating a fairly uniform vesicle population (moderate monodispersity) (Fig. 2B).

Flow cytometry analysis of 18 randomly selected samples (six from each study group) confirmed the presence of CD61 and CD83 antigens on EVs membrane (~ 14% of double positive CD63 FITC/CD81 PB objects) (Fig. 2C).

### MiRNA expression profiling

Raw sequencing data obtained from RNA sequencing underwent a comprehensive quality control assessment using FastQC (v0.12.1). Reads were subsequently filtered, trimmed, and evaluated for quality using fastp (v0.23.4). Additional miRNA-specific quality assessment was conducted with miRTrace (v1.0.1) to evaluate miRNA composition and potential biases. Processed reads were aligned to the human reference genome (GRCh38.p14/hg38) using Bowtie (v1.1.1) aligner, optimized for short reads.

Differential miRNA expression analysis was conducted using DESeq2 (v1.44.0) in R (v4.4.1) to identify statistically significant differences in miRNA expression between sample groups. To enhance miRNA identification, the miRTop (v0.4.25) tool was utilized, enabling classification and annotation of miRNA isoforms based on sequence variations.

Reference genome sequences were retrieved from the NCBI database, while miRNA sequences and annotations were obtained from the miRBase. The mature.fa and hairpin.fa files from miRBase were used as references for miRNA and hairpin structures, respectively.

Based on RNA sequencing analysis, two miRNAs (miR-98-5p and miR-760) were selected for RT-qPCR validation. Additionally, nine miRNAs (miR-155-5p, miR-326, miR-301a-3p, miR-191-5p, miR-223-3p, miR-181c-5p, miR-146a-5p, miR-23a-3p, and miR-16-5p) were chosen based on their established and/or predicted roles in regulating inflammation and neurodegeneration, as determined through a comprehensive literature review and the miRDB database of predicted miRNA-target interactions.

RT-qPCR differential miRNA expression was calculated with the comparative ∆∆Ct method, normalized to hsa-miR-451a, for RRMS and SPMS with HC as the reference population. The resulting −∆∆Ct values, representing log₂-transformed fold-change estimates, were analyzed for RRMS and SPMS differences. ∆Ct values were used to present the differential expression between HC group and RRMS and SPMS.

Table 4 presents the ∆Ct median ± IQR values for RT-qPCR analyzed miRNAs. The analysis revealed significant alterations in expression in both RRMS and SPMS patients compared to HC. Statistically significant differences were observed for miR-301a-3p, miR-181c-5p, miR-98-5p, and miR-760 (Kruskal–Wallis test, *p* < 0.05). miR-98-5p was significantly up-regulated in both RRMS and SPMS relative to HC, with greater up-regulation observed in SPMS. miR-301a-3p exhibited strong down-regulation in both disease groups, most prominently in RRMS, however, considering its low expression (> 40 Ct), this miRNA has been excluded from regression modeling and is presented descriptively. miR-760 was markedly down-regulated in RRMS but displayed a return toward baseline levels in SPMS. miR-181c-5p showed a consistent up-regulation in both RRMS and SPMS compared to HC.

**Table 4:** Differential EV-derived MiRNA expression in RRMS and SPMS patients and HC.

∆Ct value	HC (*n* = 30) [median ± IQR]	RRMS (*n* = 30) [median ± IQR]	SPMS (*n* = 30) [median ± IQR]	*p*-value (Kruskal-Wallis)
miR-155-5p	9.12 ± 6.06	10.89 ± 3.43 ↓	8.93 ± 5.68 ~	> 0.05
miR-326	15.64 ± 10.7	12.74 ± 12.09 ↑	11.33 ± 6.73 ↑	> 0.05
**miR-301a-3p**	11.71 ± 6.8	23.92 ± 12.1 ↓	13.91 ± 12.5 ↓	**< 0.0001**
miR-191-5p	6.12 ± 1.68	5.7 ± 1.16 ↑	5.88 ± 3.52 ↑	> 0.05
miR-223-3p	4.24 ± 2.23	5.22 ± 1.81 ↓	4.18 ± 2.41 ~	> 0.05
**miR-181c-5p**	14.71 ± 5.27	10.21 ± 7.06 ↑	9.2 ± 5.12 ↑	**0.0037**
miR-146a-5p	8.78 ± 9.13	8.01 ± 5.58 ↑	6.96 ± 4.74 ↑	> 0.05
miR-23a-3p	4.54 ± 1.96	4.26 ± 2.66 ↑	3.92 ± 2.41 ↑	> 0.05
miR-16-5p	6.71 ± 3.53	5.08 ± 4.79 ↑	5.39 ± 2.44 ↑	> 0.05
**miR-98-5p**	2.9 ± 4.87	-2.27 ± 3.19 ↑	-4.65 ± 4.5 ↑	**< 0.0001**
**miR-760**	-2.49 ± 5.01	1.96 ± 5.21 ↓	-1.73 ± 5.45 ~	**< 0.0001**

Arrows indicate the direction of miRNA regulation in disease groups relative to HC. ↑: miRNA up-regulated relative to HC; ↓: miRNA down-regulated relative to HC; ~: no meaningful change in miRNA expression relative to HC.

**Fig. 3:**
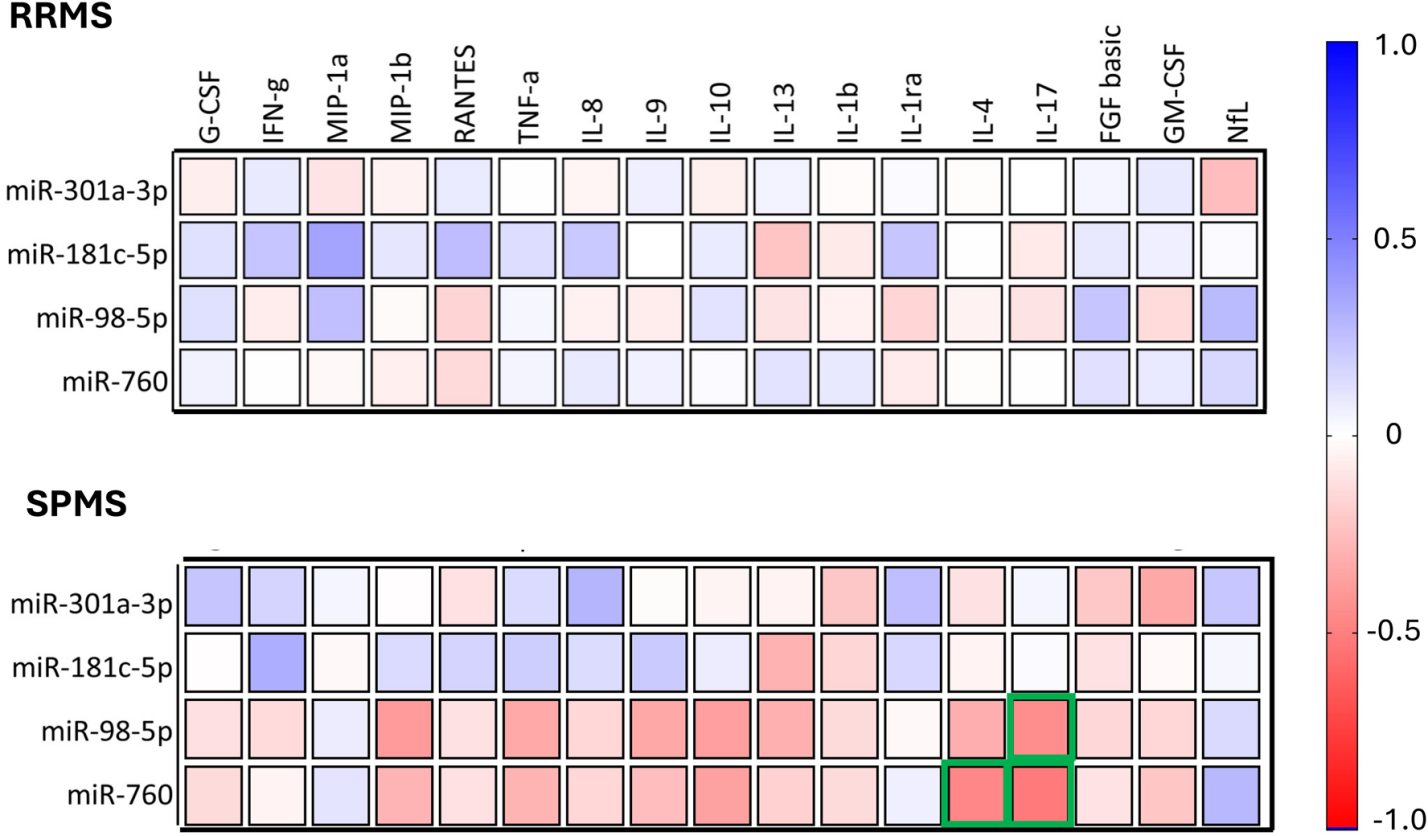
Spearman correlation between miRNA expression and protein markers in RRMS and SPMS. The color gradient scale represents the Spearman correlation coefficients (r) reflecting the strength and direction of the correlation between ΔCt values of selected miRNAs and serum cytokines, growth factors, and NfL levels in RRMS and SPMS. Values range from − 1 (negative correlation, red) to + 1 (positive correlation, blue). Statistically significant correlations (*p* < 0.05) are outlined in green.

Given the observed significant differences in both miRNA expression and protein marker levels, we next assessed their potential associations using Spearman correlation analysis (Fig. 3). In initial exploratory analysis several correlations reached nominal significance and the resulting correlation matrices revealed group-specific associations. In SPMS, miR-98-5p showed significant negative correlation with IL-17 (*p* = 0.013, *r* = -0.447); miR-760 showed significant negative correlation with IL-4 (*p* = 0.008, *r* = -0.472) and IL-17 (*p* = 0.003, *r* = -0.52). In contrast, no statistically significant correlations were observed in the RRMS group. However, no correlation remained statistically significant after FDR adjustment (q < 0.05). Therefore, these findings should be interpreted with caution and considered hypothesis-generating.

**Fig. 4:**
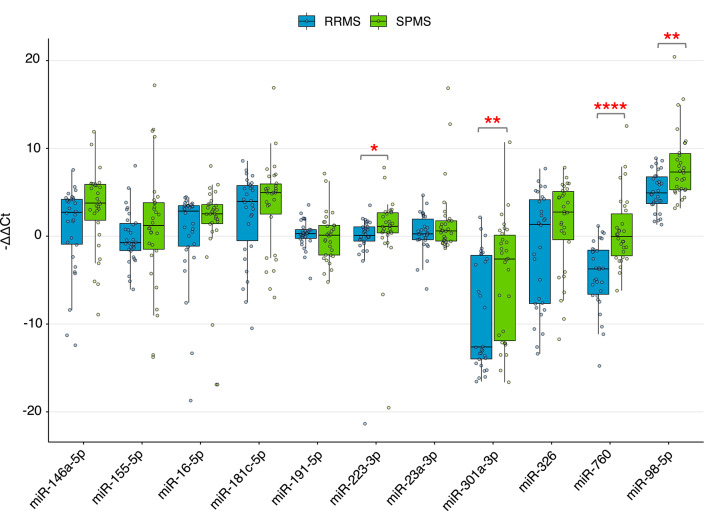
Differential miRNA expression between RRMS (*n* = 30) and SPMS (*n* = 30) measured by RT-qPCR. Individual patient values are displayed as points. Data are presented as median ± IQR of log₂-transformed fold-change (−∆∆Ct). Group differences were assessed using the Mann-Whitney U test. Significance is indicated as: *p < 0.05, **p < 0.01, and ****p < 0.0001.

Among miRNAs analyzed by RT-qPCR, four demonstrated statistically significant differences in −∆∆Ct values between RRMS and SPMS: miR-98-5p (*p* = 0.001), miR-760 (*p* < 0.0001), miR-301a-3p (*p* = 0.01), and miR-223-3p (*p* = 0.022), exhibiting the most pronounced statistical significance (Fig. 4). Of these, miR-98-5p showed marked up-regulation in both RRMS and SPMS relative to HC (log_2_FC = 5.09 and log_2_FC = 8.01). In contrast, miR-223-3p was slightly down-regulated in RRMS (log_2_FC = -0.46) and slightly up-regulated in SPMS (log_2_FC = 0.67). miR-301a-3p demonstrated a substantial down-regulation in both RRMS and SPMS (log_2_FC = -8.79 and log_2_FC = -4.72, respectively). miR-760 was markedly down-regulated (log_2_FC = -4.34) and slightly up-regulated in SPMS (log_2_FC = 0.65) compared to HC.

### Statistical modeling for stable RRMS vs. SPMS differentiation

A logistic regression model was used to assess the probability of classifying patients into the RRMS group. Based on analyzed ΔCt values, two miRNAs showed a statistically significant association with RRMS classification. Specifically, each one-unit increase in the ΔCt value of miR-146a-5p corresponded to a 26.8% increase in the odds of RRMS classification. For miR-760, a unit increase in ΔCt led to a 107.5% increase in the odds of RRMS. Statistical details are summarized in Table 5.

**Table 5:** Logistic regression analysis of MiRNA predictors for RRMS classification.

Predictor	*p*-value	Odds ratio (OR)	95% confidence interval (CI)
miR-760	< 0.0001	2.075	1.397–3.081
miR-146a-5p	0.029	1.268	1.024–1.571

**Fig. 5:**
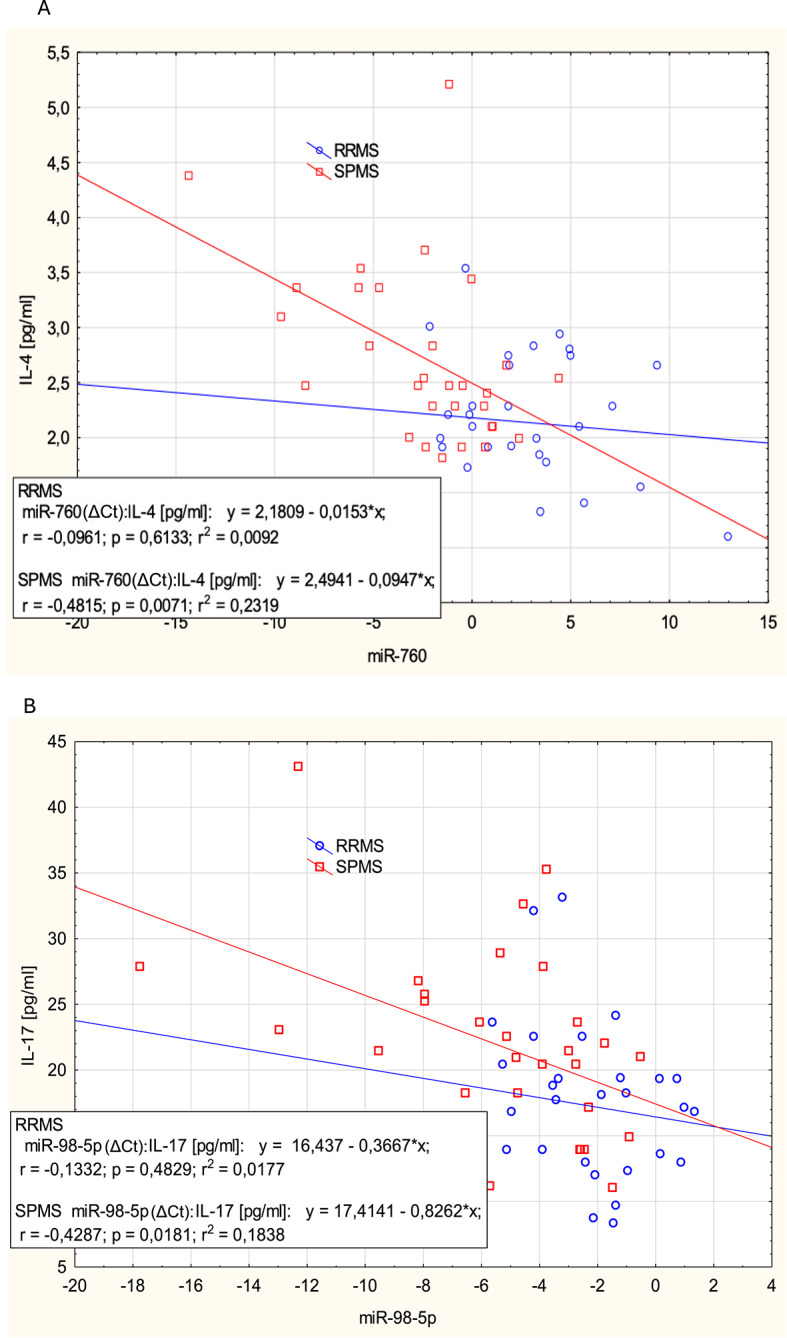
Linear regression analysis between miRNA expression (ΔCt) and cytokine levels in RRMS and SPMS patients. (**A**) Relationship between miR-760 and IL-4 levels. (**B**) Relationship between miR-98-5p and IL-17 levels. Separate regression lines are shown for RRMS and SPMS. Regression equations, correlation coefficients (r), p-values, and R² values are displayed for each group.

A linear regression analysis further revealed significant associations between specific miRNAs and immunological markers. A progressive stepwise regression approach identified a significant inverse relationship between miR-760 expression and IL-4 levels in SPMS (*p* = 0.007, *r* = -0.486, R² = 0.232), while miR-98-5p was significantly inversely associated with IL-17 levels (*p* = 0.018, *r* = -0.429, R² = 0.184) (Fig. 5). Additionally, miR-16-5p showed a significant relationship with neuronal/astroglial injury biomarkers, influencing GFAP levels in RRMS (*p* = 0.045, R² = 0.136) and NfL levels in the overall analysis (*p* = 0.036). However, upon stratification by MS subtype, the association of NfL lost statistical significance, which is consistent with the known age-dependence of this biomarker and the older age of the SPMS group.

Based on the univariate logistic regression results, the multivariate logistic regression model was developed using a forward stepwise approach. The multivariate logistic regression model, adjusted for potential collinearity, identified miR-760 as the strongest predictor of RRMS classification, followed by miR-146a-5p. Conversely, higher levels of FGF basic and miR-191-5p were associated with decreased odds of RRMS classification. Statistical details are summarized in Table 6.

**Table 6:** Multivariate logistic regression model for RRMS classification.

Predictor	*p*-value	Odds ratio (OR)	95% confidence interval (CI)
miR-760	< 0.0001	2.142	1.417–3.24
FGF-basic	0.026	0.763	0.602–0.968
miR-191-5p	0.024	0.528	0.303–0.92
miR-146a-5p	0.003	1.674	1.191–2.352

**Fig. 6:**
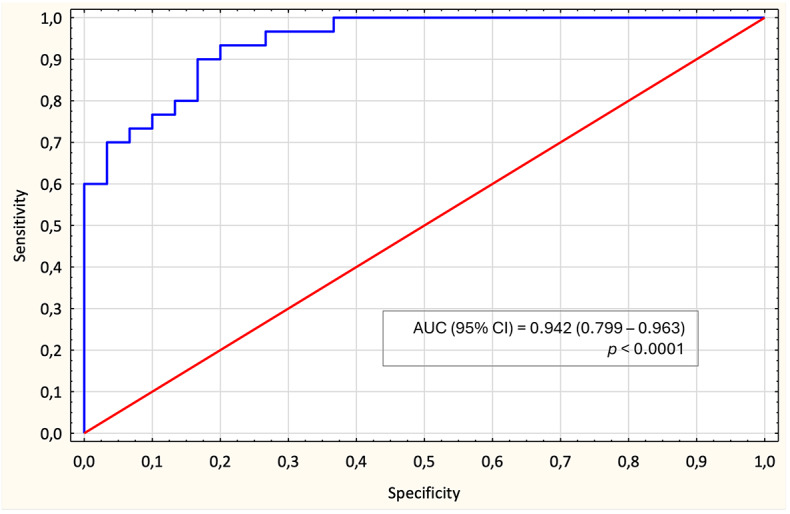
Receiver operating characteristic (ROC) curve for the multivariate model distinguishing RRMS from SPMS using fibroblast growth factor (FGF) basic, miR-760, miR-191-5p, and miR-146a-5p. The area under the curve (AUC) with 95% confidence interval (CI), and p-value were calculated. Model performance was internally validated using k-fold cross-validation.

ROC curve analysis demonstrated strong discriminatory power of the final model, yielding an AUC (95% CI) of 0.942 (0.799–0.963) (Fig. 6), confirming its robustness in distinguishing RRMS from SPMS. Goodness-of-fit analyses further supported the model’s reliability (Table 7). The Hosmer-Lemeshow (HL) test yielded a non-significant result (HL-statistic = 3.741, *p* = 0.88), indicating an adequate fit to the data. The global null hypothesis (β = 0) was tested using three methods: the likelihood ratio test (χ² = 45.78, df = 4, *p* < 0.0001), the score test (χ² = 27.51, df = 4, *p* < 0.0001), and the Wald test (χ² = 14.43, df = 5, *p* = 0.006), all of which confirmed that at least one predictor significantly contributed to the model. ROC curves of individual contributors of the multivariate model are present in Supplementary Materials (Fig. S2).

Collectively, these findings underscore the utility of miRNA expression profiles in distinguishing RRMS from SPMS, with miR-760 emerging as a particularly strong classifier.

**Table 7:** Summary of goodness-of-fit measures.

Measure	Df	Statistic	Statistic/Df
Deviance	55	37.41	0.68
Scaled deviance	55	37.41	0.68
Pearson’s Chi^2^	55	36.725	0.668
Scaled Pearson’s Chi^2^	55	36.725	0.668
AIC	–	47.41	–
AICC	–	48.521	–
BIC	–	57.882	–
Cox-Snell R^2^	–	0.534	–
Nagelkerke R^2^	–	0.712	–
Log-likelihood	–	– 18.705	–

### Target gene prediction and functional enrichment

**Fig. 7:**
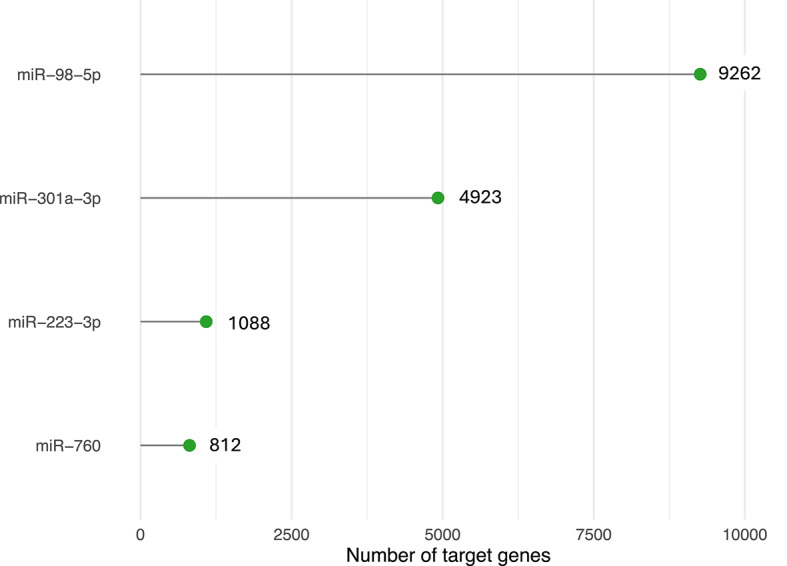
Number of experimentally validated target genes for selected miRNAs. The plot illustrates the total number of experimentally confirmed miRNA-mRNA interactions for miR-98-5p, miR-301a-3p, miR-223-3p, and miR-760. Target information was retrieved from three curated interaction databases (miRecords, miRTarBase, and TarBase). Only interactions supported by experimental evidence were included.

Human miRNA targets were retrieved from experimentally validated datasets using the multiMiR package, yielding 16 085 genes, with most targeted by miR-98-5p (Fig. 7).

**Fig. 8:**
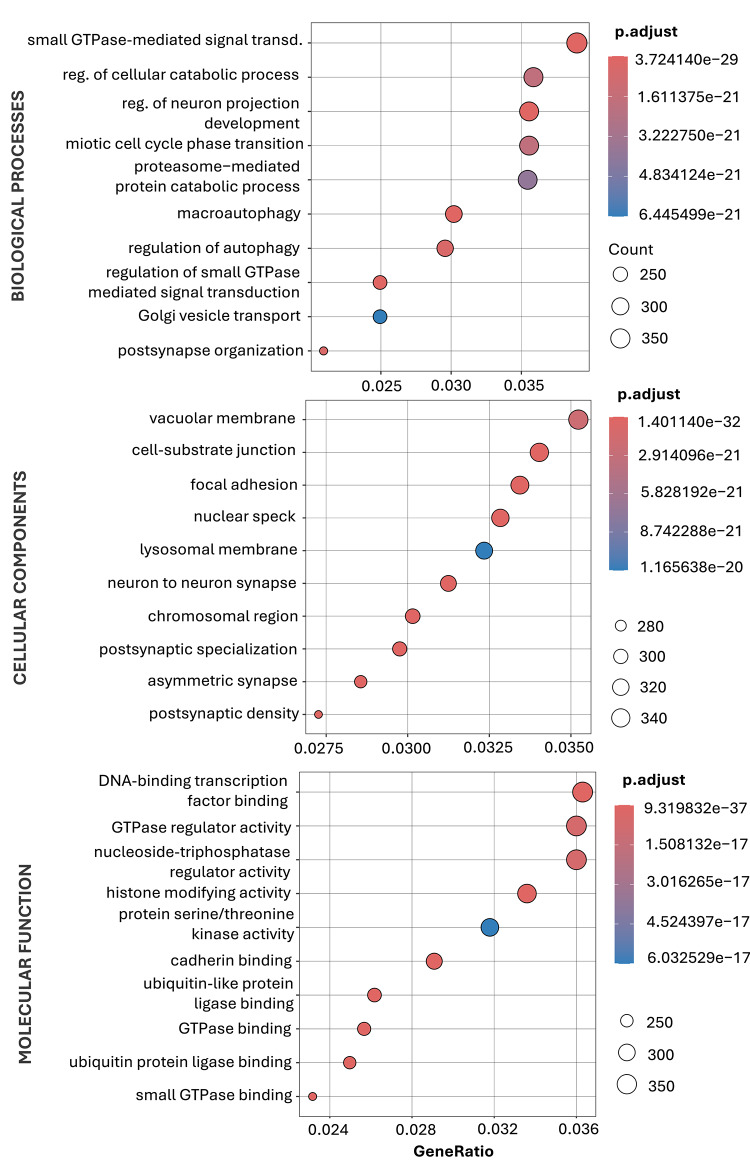
Gene Ontology (GO) enrichment analysis of target genes for miR-98-5p, miR-760, miR-301a-3p, and miR-223-3p. Bubble plots display the top enriched Biological Process, Cellular Component, and Molecular Function terms. Target genes were compiled from experimentally validated interaction databases (miRTarBase, miRecords, TarBase). Dot size corresponds to gene count, dot color indicates FDR-adjusted *p*-values (Benjamini–Hochberg), and GeneRatio represents the proportion of target genes associated with each term.

In GO functional enrichment analysis, 2 899 results were associated with differentially expressed miRNA (miR-98-5p, miR-760, miR-301a-3p, and miR-223-3p) target genes (*p* < 0.05), including 2 214 BP, 351 CC, and 334 MF. The top 10 overrepresented terms for each category are shown in Fig. 8.

The most significantly enriched BP included small GTPase-mediated signal transduction, regulation of cellular catabolic processes, neuron projection development, mitotic cell cycle phase transition, and proteasome-mediated protein catabolic processes. Additionally, pathways related to autophagy (macroautophagy, regulation of autophagy) and Golgi vesicle transport were overrepresented, indicating a strong association with intracellular signaling and degradation mechanisms. The CC analysis revealed enrichment in membrane-associated structures, including vacuolar membrane, lysosomal membrane, and cell-substrate junction, as well as synaptic components such as neuron-to-neuron synapse, postsynaptic specialization, and asymmetric synapse. Nuclear structures, including nuclear speck and chromosomal region, were also significantly represented. Significantly enriched MF included DNA-binding transcription factor binding, GTPase regulator activity, nucleoside-triphosphatase regulator activity, and histone modifying activity. Furthermore, pathways related to ubiquitin-protein ligase binding, small GTPase binding, and protein serine/threonine kinase activity were identified, highlighting the involvement of post-translational modification and intracellular signaling mechanisms.

**Fig. 9:**
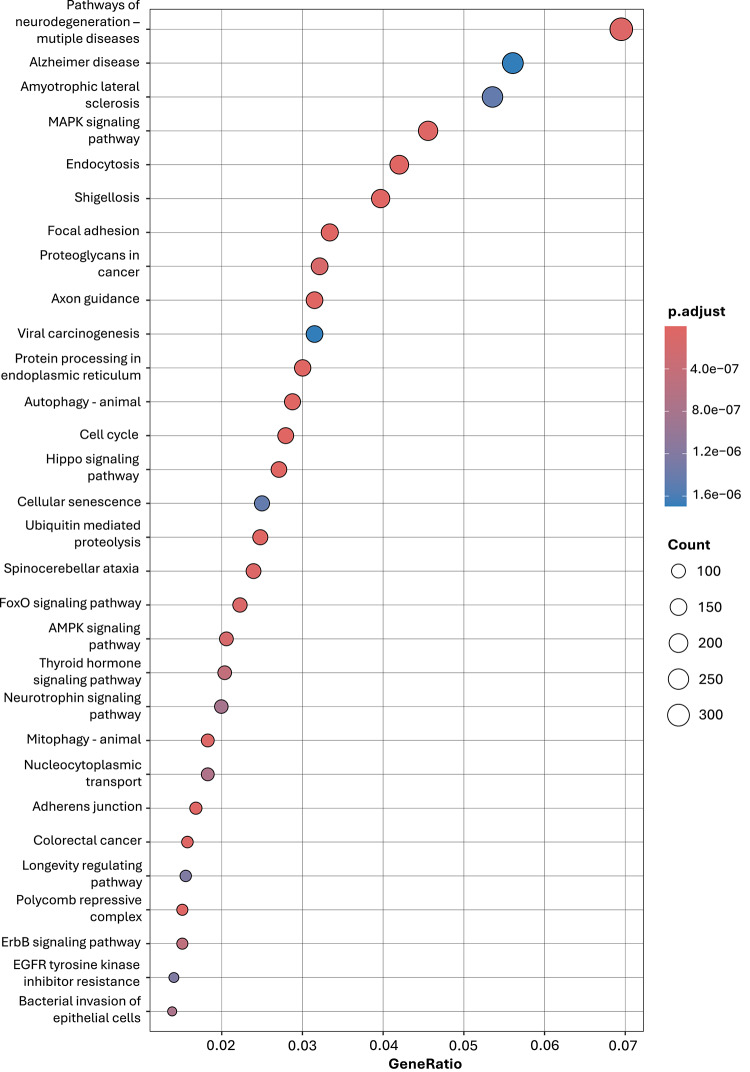
Kyoto encyclopedia of genes and genomes (KEGG) pathway enrichment analysis of target genes for miR-98-5p, miR-760, miR-301a-3p, and miR-223-3p. Bubble plot showing the top enriched KEGG pathways based on predicted and experimentally validated miRNA target genes. Dot size reflects the number of genes associated with each pathway (count), dot color indicates FDR-adjusted *p*-value, and GeneRatio represents the proportion of target genes involved in each pathway.

The KEGG^24^ pathway enrichment showed that the analyzed miRNA target genes play a role in 144 pathways. Of these, the top 30 are shown in Fig. 9. The analysis identified significant enrichment in neurodegeneration, intracellular signaling, and cellular homeostasis. Several neurodegenerative disease pathways, including Alzheimer’s disease, amyotrophic lateral sclerosis (ALS), and spinocerebellar ataxia, were overrepresented. Moreover, key intracellular signaling pathways, including MAPK, AMPK, FoxO, Hippo, and ErbB, were enriched, highlighting their roles in inflammation, cell survival, proliferation, and oxidative stress. Enrichment in autophagy, mitophagy, ubiquitin-mediated proteolysis, and protein processing in the endoplasmic reticulum indicates impaired proteostasis, a key feature in neuroinflammation. Moreover, pathways related to cellular senescence, adherens junctions, and nucleocytoplasmic transport highlight disruptions in cellular integrity and signaling.

miR-760 KEGG enrichment identified the systemic lupus erythematosus (SLE) pathway (top 1), reflecting shared autoimmune mechanisms relevant to MS. Notably, miR-760 targets included chromatin and autoantigen pathways (including histone clusters, *KMT2D*, *BRD2/4*); immune activation and signaling (*JAG1*, *MAP2K1/7*, *CDK6*); neurodegeneration and axonal support (*SDHB*, *TOMM40L*, *TPM3*); and oligodendrocyte maturation and remyelination (*WNT3*, *LIN28B*, *PYGO2*). A schematic representation of selected target pathways was provided in Supplementary Materials (Fig. S3).

**Fig. 10:**
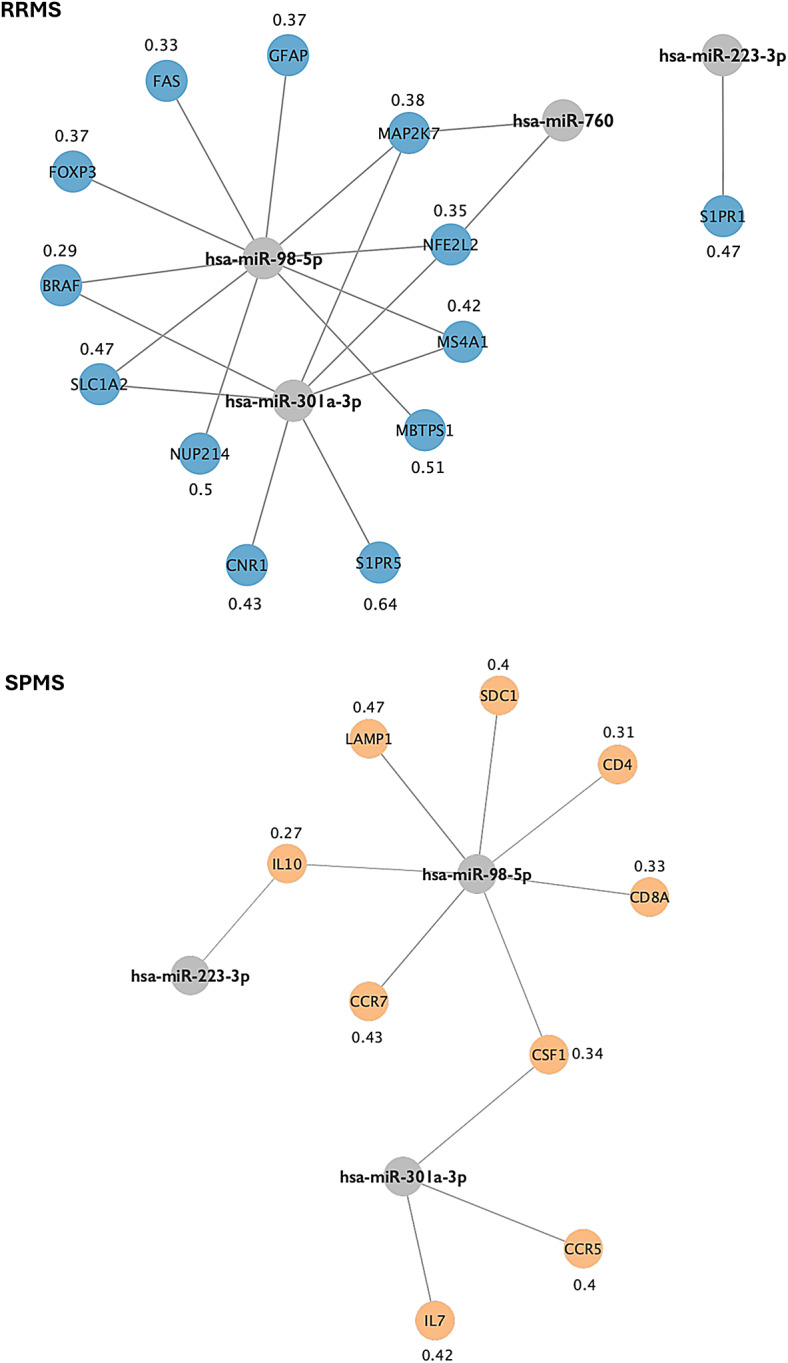
miRNA-mRNA interaction networks in stable RRMS and SPMS. Nodes represent miRNAs (sources) and their target genes, with edges indicating regulatory interactions. The values assigned to each gene indicate the disease specificity index (DSI).

To investigate molecular differences between stable RRMS and SPMS, a network analysis of miRNA-mRNA interactions was performed. The validated mRNA targets of miR-98-5p, miR-760, miR-301a-3p, and miR-223-3p were integrated with disease-associated genes retrieved from the DisGeNET database. The constructed networks present distinct regulatory patterns in stable RRMS and SPMS, with an assigned disease specificity index (DSI), whose value is inversely proportional to the number of diseases associated with a particular gene (Fig. 10). DSI in DisGeNET dataset reflects how specifically a gene is associated with a given disease, integrating information on the number and diversity of disease associations.

In RRMS, functional categories of selected genes included immune signaling (*FOXP3*, DSI = 0.37), apoptosis (*FAS*, DSI = 0.33), sphingolipid signaling (*S1PR1*, DSI = 0.47; *S1PR5*, DSI = 0.64), and stress response marker *MAP2K7* (DSI = 0.38). The astroglial injury biomarker gene *GFAP* (DSI = 0.37) was also identified. In SPMS, interactions were mainly linked to immune regulation and were targeted by miR-98-5p and miR-301a-3p: *IL10* (DSI = 0.27), *CSF1* (DSI = 0.34), *CD8A* (DSI = 0.33), and *IL7* (DSI = 0.42). Additionally, chemokine receptors involved in signal transduction, *CCR7* (DSI = 0.43) and *CCR5* (DSI = 0.4), were targeted. *SDC1* (DSI = 0.4), associated with extracellular matrix organization and hemostasis, was also identified.

These findings indicate immune-dominant regulation in SPMS, while stable RRMS exhibits additional pathways related to transcriptional control, apoptosis, and cellular stress response. miR-98-5p exhibited the highest number of interactions across both conditions, predominantly regulating immune-related genes in SPMS and extending to transcriptional and stress response pathways in stable RRMS.

## Discussion

MS is a neurodegenerative, incurable disease manifested by the destruction of myelin sheaths in result of the autoimmune mechanism, driven by infiltration of T and B-cells into the CNS^4^. Early differentiation between clinical phenotypes, particularly RRMS and SPMS, is crucial for prognosis and treatment planning, highlighting the need for reliable and easily accessible biomarkers. Although MRI of the brain and spinal cord plays a pivotal role in the diagnostic process, it does not fully reflect the picture of disease pathophysiology. To complement neuroimaging and enhance clinical decision-making, fluid-based biomarkers are extensively studied^6^. Among available biofluids, CSF stands out due to its direct relationship with the CNS, however, its collection requires an invasive procedure of lumbar puncture. Recent advances enabling the detection of brain-derived proteins, such as GFAP, CHIT1, CHI3L1, sTREM2, and NfL, in peripheral blood have opened promising avenues for non-invasive monitoring of MS pathophysiology using blood-based biomarkers^30–34^. Thus, profiling circulating EV-derived miRNAs may provide greater specificity than peripheral blood sample analysis as they are physically separated from body fluids, reflect the current molecular condition of the origin cell, and readily cross the BBB into the circulation.

In this exploratory study, we applied multi-omics approach, including cytokine profiling, neuronal and astroglial damage biomarkers measurement, EV-derived miRNA expression analysis, and integrative bioinformatics, to identify peripheral signatures that differentiate stable RRMS and SPMS phenotypes.

Among investigated EV-associated miRNAs, four candidates (miR-98-5p, miR-760, miR-301a-3p, and miR-223-3p) exhibited significantly higher expression in SPMS comparing to stable RRMS (−∆∆Ct).

Importantly, logistic regression analyses underscored miR-760 as the most promising candidate among analyzed molecules for distinguishing stable RRMS from SPMS. This finding highlights miR-760 as a potential biomarker, for stratifying patients according to disease phenotypes, pending further validation, To the best of our knowledge, this observation is novel in the MS context, considering limited previous explorations specifically addressing the clinical utility of miR-760. Importantly, hsa-miR-760-3p is the predominant and functionally active isophorm, whereas hsa-miR-760-5p is expressed at very low levels and is rarely detected in biofluids. In this study, miR-760 corresponds to miR-760-3p.

While several miRNAs have been consistently implicated in MS pathophysiology – including miR-155, miR-146a, miR-223, and miR-326 – largely in relation to immune dysregulation, Th17/Treg imbalance, microglial activation, and NF-κB-mediated inflammatory pathways – miR-760 has not been previously reported in MS.

Current evidence suggests that miR-760-3p may represent a less explored regulatory layer in MS biology, acting through neuroprotective and repair-associated mechanisms with a potential immunomodulatory activity. Previous studies have shown that miR-760-3p promotes remyelination by inhibiting G protein-coupled receptor (GPR)17, whose pathological overexpression limits late-stage myelin maturation in oligodendrocyte precursor cells^35^. Moreover, delivering exosome-encapsulated miR-760-3p to the brain has shown an anti-ferroptotic effect on neurons after ischemic brain injury^36^. This suggests that miR-760-3p may play a broader neuroprotective role beyond remyelination, potentially by modulating oxidative stress pathways and cell death mechanisms. Recent findings also indicate that reduction of miR-760-3p results in up-regulation of MAPK3K8, leading to activation of pro-inflammatory NF-κB pathway, which may support the survival and activation of autoreactive B-cells within the CNS^37^. In our analysis, both linear regression (*p* = 0.007, *r* = -0.486, R² = 0.232) and Spearman correlation (*p* = 0.008, *r* = -0.472) demonstrated a statistically significant inverse relationship between miR-760 expression and IL-4 levels in SPMS, suggesting a potential immunoregulatory role of miR-760 in the progressive disease stage. This association was not observed in RRMS, indicating that the functional relevance of miR-760 may be phenotype-specific. Taken together, these findings support the hypothesis that miR-760 may contribute to MS-related neuroimmune processes in a manner that is partially distinct from extensively studied inflammatory miRNAs, while underscoring the need for dedicated mechanistic studies and independent cohort validation.

Notably, a recent immunophenotyping study reported that while a range of Th17-lineage subsets is expanded in RRMS, only Th17 cells show increased frequencies in SPMS, supporting the involvement of Th17-driven inflammation in progressive disease stages^38^.

Given the central role of Th17 cells in MS immunopathology^39^, other miRNAs associated with this T-cell subset are of particular interest. Among them, miR-98-5p, exerts a protective effect by preventing BBB dysfunction and inhibiting neuroinflammation in the CNS^40–43^. Dysregulation of miR-98-5p has been reported in several MS studies^44–46^. Moreover, its significant correlation with IL-17 levels supports emerging evidence that identifies miR-98-5p as a crucial regulator of inflammatory pathways, particularly via modulation of Th17 responses^47,48^. In our study, stepwise linear regression revealed a significant inverse association between miR-98-5p and IL-17 levels in SPMS (*p* = 0.018, *r* = -0.429, R² = 0.184). This finding was confirmed by Spearman correlation analysis (*p* = 0.013, *r* = -0.447), with the association again observed exclusively in SPMS patients. These results suggest a stage-specific role for miR-98-5p in modulating Th17-related inflammation. Our findings are consistent with previous studies showing that miR-98-5p inhibits Th17 cells differentiation in experimental autoimmune encephalomyelitis (EAE) – likely through the direct or indirect down-regulation of RAR-related orphan receptor gamma t (RORγt), the key transcription factor driving Th17 cell development^47^.

miR-301a-3p is a Th17 subset-associated miRNA with possible functional role in immune alterations, autoimmune demyelination, and neurodegeneration^49–52^. Although miR-301a-3p appeared differentially expressed in ΔCt values in our study, the Ct levels indicate very low expression, therefore, this miRNA was excluded from predictive modeling. In other study, exosomal miR-301a-3p was decreased in RRMS patients during relapse and proposed as a potential relapse biomarker^53^. Nevertheless, research results are not consistent in this matter – peripheral blood mononuclear cells (PBMCs)-derived miR-301a expression was found up-regulated in RRMS patients in relapse^54^ and higher in post-acute vs. stable phase of remission^55^. This inconsistency underscores also the need for further studies to clarify the temporal and cellular context of miR-301a-3p expression during disease activity and also highlights the importance of sample origin in interpreting molecular signatures in MS. A recent MS study suggested that miR-223-3p could be a therapeutic target for chronic inflammation by improving the immunosuppressive function of myeloid-derived suppressor cells via a STAT3-dependent mechanism^56^. Here, we found its expression to be significantly up-regulated in SPMS compared to stable RRMS, extending recent findings on its potential as a differential biomarker of neurodegenerative conditions, a regulator of MS-related processes, including Th1 differentiation, macrophage M2 polarization, and myelin debris clearance, and biomarker distinguishing MS phenotypes^57–61^. Importantly, miR-223-3p has been found up-regulated at sites of myelin damage in both MS and experimental model of demyelination and identified as an endogenous regulator of the NLRP3 (NBD-, LRR- and pyrin domain-containing protein 3) inflammasome^62^. By targeting NLRP3, miR-223-3p may help limit the production of pro-inflammatory cytokines such as IL-1β and IL-18, thereby reducing chronic microglial activation and subsequent tissue damage. Moreover, miR-223-3p expression was negatively correlated with T1 lesion volumes in SPMS and PPMS, and its temporal variability was associated with relapse phases^63^. The inverse correlation with lesion burden implies a potential neuroprotective or inflammation-limiting role of miR-223-3p, particularly in progressive MS forms. Furthermore, its temporal fluctuations during relapse may support its use in monitoring disease activity or therapeutic response over time.

Our multivariate logistic regression integrating FGF basic, miR-760, miR-191-5p, and miR-146a-5p demonstrated excellent discriminatory power in differentiating stable RRMS from SPMS. The ROC curve analysis yielded an AUC of 0.942 (Fig. 6). Studies have shown the high expression of FGF basic within the neuroinflammatory lesions and positive correlation with macrophages and microglia activation. In contrast, in the myelin oligodendrocyte glycoprotein (MOG)_35-55_-induced EAE, a commonly used animal model of MS, the level of FGF basic was reduced, suggesting its involvement in promoting remyelination^64^. This effect was mediated through ERK/Akt phosphorylation, brain-derived neurotrophic factor (BDNF), and the down-regulation of remyelination inhibitors^57^. miR-191-5p, though less studied in MS, was found up-regulated both in SPMS and PPMS^65^. Evidence regarding the diagnostic or phenotypic utility of miR-191-5p in MS remains inconsistent. Some studies have reported no significant differences in its serum levels between MS patients and healthy individuals, nor between RRMS and progressive MS subtypes^66^; while others observed its overexpression in RRMS and PPMS, with no differences between the subtypes^67^. Interestingly, a negative correlation was observed between miR-191-5p expression and disease duration, suggesting a possible link to long-term disease progression^66^. This finding may indicate a role for miR-191-5p in the late stage of MS, consistent with its proposed value as a predictive biomarker for Alzheimer’s disease development^68^. In contrast to the ambiguous role of miR-191-5p, miR-146a-5p is one of the most extensively studied and well-characterized inflammation-related miRNAs in MS. Acting as a suppressor of innate immune activation it has been consistently implicated in diagnostic and prognostic contexts, showing strong potential as a biomarker for disease activity and progression^69^.

Functional enrichment analysis of the predicted miRNA targets revealed several key biological processes and signaling pathways that may underlie the molecular divergence between stable RRMS and SPMS. Among the most significantly overrepresented were small GTPase-mediated signaling, autophagy, cellular senescence, and pathways implicated in major neurodegenerative diseases, including Alzheimer’s and ALS. These findings align with recent data suggesting impaired autophagic flux and defective protein homeostasis as critical drivers of neurodegeneration^70,71^. Furthermore, the marked enrichment of key intracellular signaling pathways (such as MAPK, AMPK, FoxO, Hippo, and ErbB) known to influence neuroinflammation, oxidative stress responses, and neuronal survival suggests that miRNA-regulated disruption of cellular homeostasis may play a pivotal role in SPMS progression^72–80^.

Network analyses revealed distinct miRNA-mRNA regulatory architectures associated with each MS phenotype, reflecting divergent underlying mechanisms of the disease. In SPMS, regulatory networks were dominated by immune-related genes primarily targeted by miR-98-5p and miR-301a-3p, including *IL10*, *CSF1*, *CD8A*, and chemokine receptors *CCR5* and *CCR7* – previously implicated in chronic neuroinflammation, impaired immune resolution, and sustained microglial activation^81–87^. This supports growing evidence that progressive MS is driven by compartmentalized, smoldering inflammation within the CNS^88^. In turn, the stable-specific network revealed a broader functional diversity, involving pro-apoptotic signaling (*FAS*), lipid-mediated immunoregulation (*S1PR1*, *S1PR5*), and stress-response pathways (*MAP2K7*)^89–91^.

Despite the promising findings, several limitations of this study should be acknowledged. Although patients had not received corticosteroids for at least three weeks prior to sampling, an interval of at least one month is recommended for transcriptional and cytokine studies. Thus, residual effects of corticosteroid treatment cannot be entirely excluded. Moreover, the relatively moderate sample size may limit the generalizability of the results and warrants validation in larger independent cohorts. It should be also noted that although EVs isolation and characterization protocols were performed using TEM, flow cytometry, and ZetaSizer analysis, Western Blotting and nanoparticle tracking analysis (NTA) were not included. Future work incorporating these methods will further enhance EV purity assessment and biomarker precision. Moreover, the RNA-seq discovery cohort included a limited number of samples, which constrains statistical power and generalizability. Therefore, the sequencing results should be considered exploratory. While the expression of miRNAs was analyzed by RT-qPCR, an external validation is warranted. Further studies integrating functional validation and independent patient cohorts will be key to translating these observations into clinically relevant biomarkers.

Collectively, our findings extend the current understanding of MS phenotype differentiation, underscoring plasma-derived EV-miRNAs as putative biomarkers with potential translational relevance. Notably, miR-760 and miR-98-5p represent particularly important discoveries within this study. They were initially identified through RNA-sequencing and subsequently validated by RT-qPCR, and demonstrated statistically significant associations with molecular disease parameters, including IL-4 and IL-17 levels. These findings were further supported by group-specific correlation matrices, which revealed that selected miRNA–cytokine relationships were present exclusively in the SPMS group. Their expression profiles and integration into predictive logistic regression models point to their potential relevance as indicators of immune activity of MS subtypes. Nevertheless, these findings warrant further validation in independent, longitudinal cohorts and functional studies to establish their clinical applicability and mechanistic significance.

## Supplementary Information

Below is the link to the electronic supplementary material.

Supplementary Material 1

## Data Availability

The raw and processed RNA sequencing data generated in this study have been deposited in the Gene Expression Omnibus (GEO) under accession number GSE303912.To review GEO accession GSE303912:Go to (https:/www.ncbi.nlm.nih.gov/geo/query/acc.cgi?acc=GSE303912) Enter token oxufugusxnobngf into the box.

## Abbreviations

ALS: Amyotrophic lateral sclerosis
AUC: Area under the curve
BBB: Blood-brain barrier
BDNF: Brain-derived neurotrophic factor
BP: Biological processes
CC: Cellular components
cDNA: Complementary DNA
CI: Confidence interval
CIS: Clinically isolated syndrome
CNS: Central nervous system
CPDA: Citrate phosphate dextrose adenine
CRP: C-reactive protein
CSF: Cerebrospinal fluid
CV: Coefficient of variation
DLS: Dynamic light scattering
DMTs: Disease-modifying therapies
DSI: Disease specificity index
EAE: Experimental autoimmune encephalomyelitis
EDSS: Expanded disability status scale
ELISA: Enzyme-linked immunosorbent assay
ESR: Erythrocyte sedimentation rate
EV: Extracellular vesicle
FC: Fold-change
FDR: False discovery rate
FGF: Fibroblast growth factor
G-CSF: Granulocyte colony-stimulating factor
GDA: Disease-gene association
GFAP: Glial fibrillary acidic protein
GM-CSF: Granulocyte macrophage colony-stimulating factor
GO: Gene ontology
GPR: G protein-coupled receptor
HC: Healthy control
HL: Hosmer-Lemeshow
IFN: Interferon
IL: Interleukin
IP: Interferon gamma-induced protein
IQR: Interquatrile range
ISEV: International society of extracellular vesicles
KEGG: Kyoto encyclopedia of genes and genomes
MCP: Monocyte chemoattractant protein
MF: Molecular functions
MIP: Macrophage inflammatory protein
miRNA: microRNA
MOG: Myelin oligodendrocyte glycoprotein
MOGAD: Myelin oligodendrocyte glycoprotein antibody-associated disease
MRI: Magnetic resonance imaging
MS: Multiple sclerosis
NfL: Neurofilament light chain
NLRP3: NBD-, LRR- and pyrin domain-containing protein 3
NMOSD: Neuromyelitis optica spectrum disorder
OD: Optical density
PBMCs: Peripheral blood mononuclear cells
PBS: Phosphate-buffered saline
PDGF: Platelet-derived growth factor
PDI: Polydispersity index
PPMS: Primary progressive multiple sclerosis
RANTES: Regulated on activation, normal T expressed and secreted
ROC: Receiver operating characteristic
RORγt: RAR-related orphan receptor gamma t
RRMS: Relapsing-remitting multiple sclerosis
RT: Room temperature
RT-qPCR: Quantitative real-time PCR
SPMS: Secondary progressive multiple sclerosis
TEM: Transmission electron microscopy
Th: T helper
TNF: Tumor necrosis factor
Treg: T regulatory
VEGF: Vascular endothelial growth factor
