# Supplementary material for: Extracellular vesicle-derived miR-760 as a novel promising candidate biomarker differentiating stable RRMS from SPMS
Source: Sci Rep. 2026 Jan 14;16:5208. doi: 10.1038/s41598-026-35189-y (PMC12881579; doi:10.1038/s41598-026-35189-y)
Supplement: Supplementary file 1 — Supplementary Material 1 [file 41598_2026_35189_MOESM1_ESM.docx]

**Supplementary Materials**

**Table S1.** Minimal expected concentrations for individual cytokines measured in the Bio-Plex multiplex assay. The acceptable limits for intra-assay and inter-assay coefficients of variation (CV%) were <15% and <20%, respectively.

| **Biomarker** | **Minimal expected concentration [pg/mL]** | **Intra-assay CV% for positive control (plate 1)** | **Intra-assay CV% for positive control (plate 2)** | **Inter-assay CV% for positive control** |
| --- | --- | --- | --- | --- |
| **IL-1b** | 0.08 | 1.28 | 1.24 | 6.72 |
| **IL-1ra** | 2.35 | 5.27 | 1.13 | 0.88 |
| **IL-2** | 0.29 | 1.77 | 1.17 | 5.44 |
| **IL-4** | 0.08 | 2.77 | 1.27 | 7.57 |
| **IL-5** | 1.87 | 0.07 | 1.26 | 1.61 |
| **IL-6** | 0.09 | 3.81 | 0.91 | 5.01 |
| **IL-7** | 0.68 | 2.87 | 1.23 | 1.9 |
| **IL-8** | 0.23 | 5.8 | 1.09 | 4.31 |
| **IL-9** | 0.52 | 2.85 | 1.19 | 2.62 |
| **IL-10** | 0.27 | 1.59 | 1.26 | 0.78 |
| **IL-12** | 0.47 | 0.71 | 1.25 | 4.63 |
| **IL-13** | 0.06 | 4.09 | 1.13 | 10.64 |
| **IL-15** | 4.87 | 1.09 | 1.16 | 3.35 |
| **IL-17** | 0.71 | 3.46 | 0.89 | 4.05 |
| **eotaxin** | 0.02 | 2.17 | 0.88 | 14.9 |
| **FGF basic** | 1.03 | 3.08 | 0.98 | 8.85 |
| **G-CSF** | 1.72 | 4.21 | 0.92 | 1.12 |
| **GM-CSF** | 0.1 | 0.14 | 1.09 | 0.88 |
| **IFN-γ** | 0.28 | 0.33 | 1.02 | 2.51 |
| **IP-10** | 0.42 | 0.74 | 1.16 | 4.96 |
| **MCP-1** | 0.13 | 0.31 | 0.95 | 1.65 |
| **MIP-1α** | 0.02 | 1.75 | 0.66 | 9.35 |
| **PDGF-bb** | 0.82 | 0.32 | 1.3 | 1.18 |
| **MIP-1β** | 0.12 | 0.75 | 1.03 | 2.92 |
| **RANTES** | 0.24 | 7.06 | 1.26 | 5.37 |
| **TNF-α** | 0.95 | 1.82 | 1.21 | 6.74 |
| **VEGF** | 1.5 | 5.87 | 1.18 | 8.00 |

**Table S2.** Lower limits of detection for NfL and GFAP biomarkers measured by ELISA. The acceptable limit for the intra-assay coefficient of variation (CV%) was <15%.

| **Biomarker** | **Lower limit of detection [pg/mL]** | **Intra-assay CV% [median ± IQR]** |
| --- | --- | --- |
| **GFAP** | 0.57 | 7.56 ± 10.2 |
| **NfL** | 9.38 | 10.82 ± 16.32 |

**Table S3.** Ct values of miRNAs in healthy controls (HC), relapsing–remitting multiple sclerosis (RRMS), and secondary progressive multiple sclerosis (SPMS) patients. Data are presented as median ± IQR.

| **Ct value** | **HC (n = 30)**  **[median ± IQR]** | **RRMS (n = 30)**  **[median ± IQR]** | **SPMS (n = 30)**  **[median ± IQR]** |
| --- | --- | --- | --- |
| **miR-155-5p** | 32.55 ± 5.5 | 32.56 ± 4.95 | 32.26 ± 4.01 |
| **miR-326** | 41.55 ± 13.33 | 35.26 ± 13.62 | 37.34 ± 11.53 |
| **miR-301a-3p** | 34.45 ± 7.67 | 46.9 ± 14.47 | 36.62 ± 4.77 |
| **miR-191-5p** | 31.25 ± 5.38 | 28.99 ± 3.34 | 31.66 ± 4.97 |
| **miR-223-3p** | 28.95 ± 6.28 | 29.06 ± 4.23 | 28.77 ± 4.5 |
| **miR-181c-5p** | 37.7 ± 8.32 | 32.75 ± 8.12 | 33.72 ± 9.77 |
| **miR-146a-5p** | 33.0 ± 13.48 | 31.58 ± 8.07 | 30.62 ± 7.27 |
| **miR-23a-3p** | 29.25 ± 4.3 | 27.19 ± 3.55 | 28.03 ± 3.43 |
| **miR-16-5p** | 32.25 ± 8.3 | 29.97 ± 8.0 | 28.91 ± 6.1 |
| **miR-98-5p** | 26.75 ± 3.68 | 20.63 ± 1.55 | 19.43 ± 1.65 |
| **miR-760** | 22.65 ± 1.07 | 25.48 ± 3.06 | 22.62 ± 1.09 |

**Fig. S1**. Distribution of ΔCt values for the endogenous control (miR-451a) across all plasma samples. Data are presented as median with interquartile range (IQR).

**
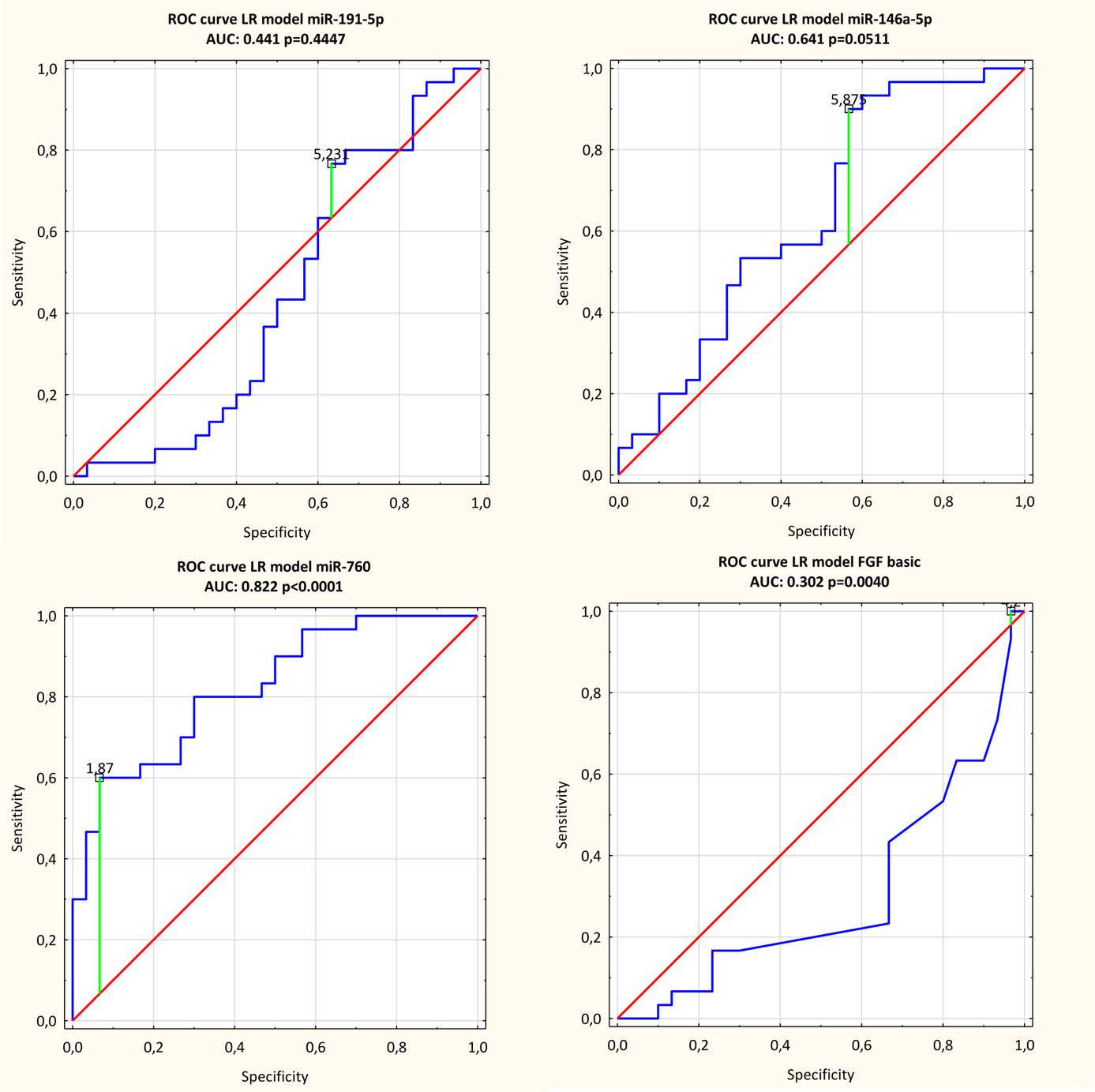
**

**Fig. S2.** Receiver operating characteristic (ROC) curve analyses of individual contributors to the multivariate model distinguishing stable RRMS from SPMS. The area under the curve (AUC) and corresponding p-values are indicated for each variable. The optimal cut-off point, determined using the Youden index, is marked in green.

**Fig. S3.** **Bioinformatic overview of putative miR-760 molecular functions.** Integrated analysis of experimentally validated and predicted miR-760 targets revealed four major biological modules relevant to MS pathophysiology: chromatin and autoantigen pathways, immune activation signaling, neurodegeneration and axonal integrity, and oligodendrocyte maturation and remyelination.
